# Loss of Social/Non-social Context Discrimination by Movement Acceleration in the Valproate Model of Autism

**DOI:** 10.3389/fnbeh.2020.555610

**Published:** 2021-01-11

**Authors:** Nelva T. Quezada, Sebastiana F. Salas-Ortíz, Francisco A. Peralta, Felipe I. Aguayo, Katherine P. Morgado-Gallardo, Catherine A. Mac-Rae, Jenny L. Fiedler, Esteban E. Aliaga

**Affiliations:** ^1^Department of Kinesiology, Faculty of Health Sciences, Universidad Católica del Maule, Talca, Chile; ^2^Laboratory of Neuroplasticity and Neurogenetics, Department of Biochemistry and Molecular Biology, Faculty of Chemical and Pharmaceutical Sciences, Universidad de Chile, Santiago, Chile; ^3^Department of Psychology, Faculty of Health Sciences, Universidad Católica del Maule, Talca, Chile; ^4^Faculty of Health Sciences, The Neuropsychology and Cognitive Neurosciences Research Center (CINPSI-Neurocog), Universidad Católica del Maule, Talca, Chile

**Keywords:** autism, valproate, social behavior, acceleration, movement kinematics

## Abstract

Autism spectrum disorder (ASD) is a neurodevelopmental alteration characterized by social/communicative deficits, repetitive/stereotyped movements, and restricted/obsessive interests. However, there is not much information about whether movement alterations in ASD comprise modifications at the basic kinematic level, such as trajectory and velocity, which may contribute to the higher level of processing that allows the perception and interpretation of actions performed by others, and hence, impact social interaction. In order to further explore possible motor alterations in ASD, we analyzed movement parameters in the Valproate (VPA) animal model of autism. We found that VPA-treated rats displayed greater movement acceleration, reduced distance between stops, spent more time in the corner of the open-field arena, and executed a number of particular behaviors; for example, supported rearing and circling, with no major changes in distance and velocity. However, in the social interaction test, we found other alterations in the movement parameters. In addition to increased acceleration, VPA-rats displayed reduced velocity, increased stops, reduced distance/stop and lost the social/non-social area discrimination that is characteristic of control rats in acceleration and stops variables. Hence, even if prenatal VPA-treatment could have a minor effect in motor variables in a non-social context, it has a crucial effect in the capacity of the animals to adjust their kinematic variables when social/non-social context alternation is required.

## Introduction

Autism spectrum disorder (ASD) is a multifactorial group of neurodevelopmental alterations of unknown etiology, in which genetic and environmental factors are involved (De Rubeis et al., 2014; Modabbernia et al., 2017). This condition elicits three principal behavioral alterations: (i) social and communicative deficits, (ii) repetitive/stereotyped movements, and (iii) restricted/obsessive interests (Frith and Happé, 2005). The diversity of these altered behavioral features supports a concept in which dysfunctions underlying ASD are produced by atypical neurodevelopmental trajectories, leading to a misconnected brain, where over-connected and under-connected circuits are developed (Wass, 2011). Moreover, growing evidence considered ASD as a multisystemic disorder affecting other pathophysiological domains, such as the gut-intestinal, immunologic, metabolic and motor systems (Bhat et al., 2011; Careaga and Ashwood, 2012; Julio-Pieper et al., 2014; Cheng et al., 2017; Sciara et al., 2020).

Motor alterations in ASD were described in the earliest studies of this pathology by Kanner (1943) and Asperger (1944), but despite the existence of stereotyped movements, these do not form part of the current diagnostic criteria. However, these alterations could be early indicators of a deviant neurodevelopment that precedes the emergence of social and communicative deficits. ASD alters motor control in several domains, such as posture, gait, and praxis, producing decreased static and dynamic postural control during quiet stance (Fournier et al., 2010a,b); gait alterations that may involve cerebellar or basal-ganglia frontostriatal circuits (Rinehart et al., 2006a,b), delayed development of anticipatory control (Schmitz et al., 2003; Hobson et al., 2009) and an important deficit in motor coordination (Fournier et al., 2010a,b). In addition, alterations in praxis have also been reported and suggest a deficit in the acquisition of internal motor models and hence, altered motor learning (Gidley Larson et al., 2008; Cossu et al., 2012). Importantly, evidence shows that praxis errors correlate with the Autism Diagnostic Observation Schedule (ADOS) score (Dziuk et al., 2007; Dowell et al., 2009). Furthermore, the wide range of motor impairments in ASD, linked to its persistent nature (Van Waelvelde et al., 2010), led to the proposal that motor dysfunction is rather a core feature of ASD, and not just a simple symptom (Rinehart and McGinley, 2010). In agreement with this hypothesis, movement execution in ASD displays atypical kinematics, which additionally is well correlated with the ADOS score (Cook et al., 2013).

Furthermore, animal models of autism have allowed us to explore the cellular and molecular underlying mechanisms and, to some extent, the associated motor alterations (Peralta et al., 2016; Fuentealba et al., 2019). In particular, prenatal exposition to the anticonvulsant drug Sodium Valproate (VPA) is currently the best validated model for environmentally induced ASD (Rodier et al., 1996; Wagner et al., 2006; Mabunga et al., 2015; Tartaglione et al., 2019), where morphological anomalies have been detected in motor areas, such as the facial motor nuclei (Oyabu et al., 2013), the motor cortex (Snow et al., 2008) and the cerebellar cortex (Wang et al., 2018). However, in this model, the descriptions of motor alterations, other than stereotyped/repetitive movements, have been limited to a general delay in achieving developmental milestones, and reduced locomotion and object manipulation (Schneider and Przewłocki, 2005; Reynolds et al., 2012; Peralta et al., 2016). Interestingly, motor deficiencies in the VPA-model, as occurs in ASD individuals, correlate with social deficits (Al Sagheer et al., 2018).

In order to explore possible motor alterations in the VPA-model related to independent free exploration or social interaction, we evaluated kinematic parameters in two habitually used behavioral tests for laboratory rats: the open field test (OFT) and the three-chamber social test (3C-ST). The first one is designed to evaluate locomotor activity and exploratory behavior without social demands and the second test specifically evaluates social interaction. In brief, the aim of our study was to determine whether the VPA autism model shows kinematic particularities and if they are influenced by social or non-social contexts.

## Materials and Methods

### Animals

Pregnant female Sprague-Dawley rats were obtained from the Faculty of Chemical and Pharmaceutical Sciences, Universidad de Chile and housed in 25 × 45 × 15 cm cages under standard conditions (21 ± 2°C, 12:12 h light-dark cycle and *ad libitum* access to food and water). On embryonic day 12.5 (E12.5) experimental rats received a single i.p. injection of Sodium Valproate (450 mg/Kg, Sigma-Aldrich), while the control group received a saline solution injection. On postnatal day 21 (PN21), the day of weaning, pups were separated from their dam and males were housed in groups of 3–4 littermates until P30, when behavioral tests were performed. We only used males in this study, considering that prenatal VPA treatment produces effects both in males and females, but with qualitative and quantitative differences (Hara et al., 2017; Al Sagheer et al., 2018). At all times, efforts were made to minimize both the number of animals used and their suffering. All procedures were approved by the Ethic Committee of the Chilean Science and Technology National Commission (CONICYT), in compliance with the National Institutes of Health Guide for Care and Use of Laboratory Animals [National Research Council (NRC), 2011].

### Behavioral Tests

All tests were performed between 9:00 and 15:00 h and recorded by a video camera located at 1.5 m from the test surface. Tests were performed sequentially with an interval of 48 h, beginning with the open field test. Animals were habituated to the room 5 min before each test and the apparatus were cleaned with 5% ethanol between each testing of the animals. No other person besides the experimenter was in the room. The tests were performed with 60 db of white noise and there were no visual clues in the room.

#### OFT

A black OFT of 60 × 60 cm was used. At the beginning of the test, every animal was located at the center of the illuminated arena and allowed to freely explore; such behavior was recorded for 5 min. OFT was conducted in two batches of control and experimental rats. A first batch composed of 13 control and 7 VPA-treated rats (*n* = 7), and a second batch composed of 8 control and 8 VPA-treated rats.

#### 3C-ST

Social test was performed in a third batch of control (*n* = 8) and VPA treated (*n* = 11) rats. The apparatus consisted of three 30 × 22 cm adjoining chambers of transparent acrylic, connected by 10 × 10 cm doors. Because we used juvenile rats (PN30) we have used the apparatus dimensions for adult mice, which are similar in weight (Moy et al., 2004). Initially, the animal was allowed to habituate to the central chamber for 5 min (habituation stage) and then, the doors leading to both lateral chambers were opened. In this stage (social stage), the rats were exposed to a familiar congener located in one of the lateral chambers inside of a wired cage, while the other one contained an empty wired cage. Social interaction was recorded and evaluated during 10 min. Three VPA treated animals did not move at all during social stage of the 3C-ST, hence these animals were take-out of the social stage analysis.

#### EPM

In order to complement the behavioral evaluations, we performed the elevated plus maze test (EPM) to examine anxiety levels, as previously reported (Peralta et al., 2016) in the second batch of control and VPA-treated rats. Briefly, the arms measured 40 × 10 cm and the enclosed arms were limited by walls 30 cm high, and elevated 50 cm from the floor. At the beginning, the rat was placed in the center, facing to an open arm and video recording was performed during 5 min.

### Behavioral and Kinematic Analysis

All videos were recorded with a web video camera (C210, Logitech International S.A., Lausanne, Switzerland), converted to the grayscale AVI format, and then processed by ImageJ 1.52p software (Schneider et al., 2012)^1^ using the “threshold” and “analyze particles” functions to obtain the Cartesian position coordinates (x, y) of the rat’s “center of mass” in each video frame. For all analyzed video sequences, a visual verification of whole photograms was performed in order to correct the data, when necessary. Even though time consuming, this method guarantees no biases in point position data. Data were then transferred to an Excel worksheet (Excel Microsoft 2016, Washington, United States) and each video data set was calibrated to the actual dimensions of the testing arenas. From these data sets we calculated the displacement between two consecutive frames and, using logical functions, extracted the frames in which the rats were moving and those in which the rats remained immobile. These two variables allowed us to obtain two parameters: “traveled distance,” as the cumulative sum of frame to frame displacement, and “effective velocity,” as the total distance divided by the time during which the rat was actually moving. We used the term “effective,” rather than “mean” velocity because we calculated the velocity only when the animal moved (excluding the time when it remained immobile). “Mean acceleration” was calculated as the mean value of frame to frame positive change of velocity, only counting the frames with displacements. The parameter “stops” corresponds to the number of times that a frame-series with displacements was interrupted by, at least, one frame of immobility, from another frame-series with displacements. The parameter “distance/stop” was obtained, as the total distance divided by the total number of “stops.” These analyses were performed for both the OF and 3C-ST behavioral tests.

### Evaluation of Walking Velocity and Specific Exploratory Behaviors

Specific behaviors, such as “supported rearing” (defined as straighteners putting the two paws on the walls, Sturman et al., 2018), “returns” (180° turns) and circling behavior (360° turns) were visually evaluated in the first batch of control and experimental rats by a trained -blind to condition- operator. OFT videos of the second batch were used to determinate corner activity (four 20 × 20 cm corner quadrants), percentage of the time in central 20 × 20 cm quadrant, and to evaluate walking velocity. Walking velocity was measured in specific video-segments in which the animal performed, at least three steps in a straight-line. These isolated video sequences (45 video-sequences) were analyzed with ImageJ software and velocity was determined as the displacement in cm, directly measured in the calibrated image, and the time derived from the number of photograms, according to the time resolution of the video.

### Statistical Analysis

Statistical analyses were performed using GraphPad Prism 8.0.2 (GraphPad Software Inc., San Diego, CA, United States). The non-parametric two-tailed Mann-Whitney *U*-test was used when comparing two groups (values in the total time or total area in the tests). Multiple comparisons in minute-by-minute analysis from OFT and 3C-ST-habituation and social/non-social areas- were analyzed by two-way ANOVA, followed by Tukey (minute-by-minute analysis) or Sidak (social/non-social areas) *post hoc* tests. In all cases, a *p*-value lower than 0.05 was considered as statistically significant.

## Results

In order to analyze possible motor alterations in our VPA model, we decided to explore changes in kinematic parameters in two behavioral tests; one designed to evaluate locomotor activity (OFT), and another one usually chosen to evaluate social interaction (3C-ST).

### Kinematic Parameters During the OFT

**FIGURE 1:**
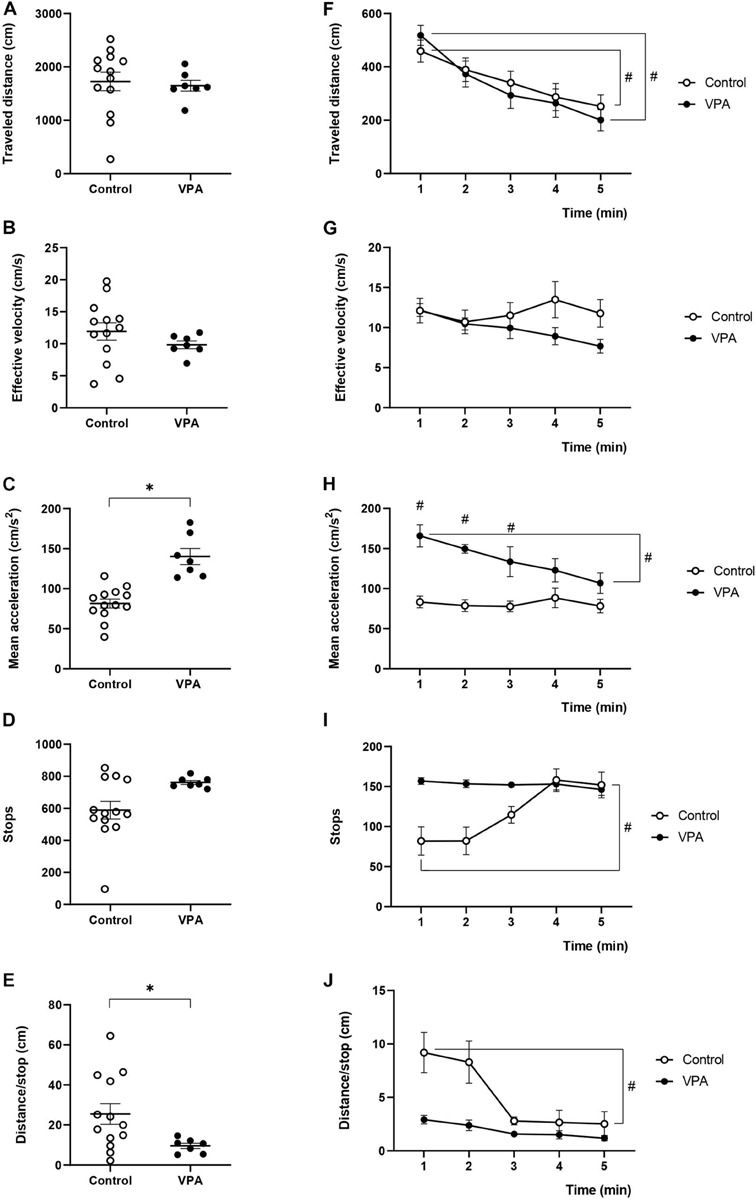
Effect of prenatal VPA treatment on kinematic parameters in the OFT. **(A)** “Traveled distance,” **(B)** “effective velocity,” **(C)** “mean acceleration,” **(D)** “stops,” and **(E)** “distance/stop” in the total duration (5 min) of the test. ^∗^*p* < 0.05; Mann-Whitney *U*-test, VPA compared to controls. Minute-by-minute analysis of **(F)** “traveled distance,” **(G)** “effective velocity,” **(H)** “mean acceleration,” **(I)** “stops,” and **(J)** “distance/stop.” ^#^*p* < 0.05; Two-way ANOVA, followed by the Tukey *post hoc* test, min 1 compared to min 5. n controls = 13, n VPA = 7.

We determined “traveled distance,” “effective velocity,” and “mean acceleration,” both in control and VPA-treated rats during the 5 min of the OFT sessions. Both groups exhibited a similar total “traveled distance” and “effective velocity” (Figures 1A,B), while the VPA-group showed increased “mean acceleration” (140.2 ± 10.9 cm/s^2^), which was higher (*p* < 0.0001, Mann-Whitney *U*-test) than that of the control group (81.3 ± 5.8 cm/s^2^) (Figure 1C). Our analysis allowed us to calculate the number of times in which the animal halted its displacement and initiated another (“stops”). Accordingly, control rats stopped 588.7 ± 57.16 times during the 5 min of the test, while the VPA-group stopped 761.71 ± 13.4 times, with no significant difference (*p* = 0.1005, Mann-Whitney *U*-test) (Figure 1D). The total traveled distance values were used to calculate the mean distance between stops: “distance/stop” for each animal. While the control group traveled a mean of 25.5 ± 5.4 cm between halts, the VPA-group traveled an average of 9.7 ± 1.5 cm, the difference was statistically significant (*p* < 0.05, Mann-Whitney *U*-test) (Figure 1E).

Nonetheless, when we analyzed the minute-by-minute data along the 5 min of the test, we detected a gradual decline for the “traveled distance” from the beginning (minute 1) to the end (minute 5). In fact, two-way ANOVA showed a significant main effect of time [*p* < 0.0001, *F*(4, 48) = 14.04], without an effect of the VPA treatment [*p* = 0.7611, *F*(1, 12) = 0.0971], or minute × VPA interaction [*p* = 0.6253, *F*(4, 18) = 0.6636], thus evidencing a reduction of the same magnitude for both control and VPA-treated groups (Figure 1F). In the control group, the “traveled distance” registered in the first minute was 459.3 ± 42.1 cm and significantly decreased at the last minute to 251.7 ± 44.9 cm (*p* < 0.01, Tukey *post hoc* test, first minute vs. last minute). Similarly, analysis in the VPA-group showed a “traveled distance” in the first minute of 517.9 ± 36.8 cm, which significantly decreased in the last minute, reaching 200.3 ± 29.8 cm (*p* < 0.01, Tukey *post hoc* test, first minute vs. last minute). Regarding the behavior of “effective velocity” along the five minutes of the OFT, we did not observe significant effects by two-way ANOVA analysis for time [*p* = 0.4214, *F*(4, 48) = 0.9913], VPA treatment [*p* = 0.2736, *F*(1, 12) = 1.317] nor interaction time × VPA [*p* = 0.2153, *F*(4, 18) = 1.609]. Control and VPA-groups showed a nearly invariable “effective velocity” along the trial period (*p* = 0.99 for control and *p* = 0.44 for the VPA-group; Tukey *post hoc* test, comparing the first and last minute in both cases) (Figure 1G). Interestingly, “mean acceleration” was relatively stable during the 5 min of the test in the control group, while the VPA-group showed a statistically significant decline. Two-way ANOVA analysis showed a significant main effect of time [*p* = 0.0114, *F*(4, 48) = 3.643], VPA treatment [*p* = 0.0003, *F*(1, 12) = 25.72] and time × VPA interaction [*p* = 0.0248, *F*(4, 18) = 3.3615]. “Mean acceleration” significantly decreased (*p* < 0.05, Tukey *post hoc* test) from the first minute (165.8 ± 14.8 cm/s^2^) to the last minute (106.9 ± 13.8 cm/s^2^), reaching values that were not statistically different from those of the control group in the last 2 min (Figure 1H).

As shown in Figure 1I, the “stops” was relatively stable during the 5 min of the test in the VPA group, while the control group started with a reduced number of stops in minutes 1 and 2, which rose to VPA values in minutes 4 and 5. The minute-by-minute analysis of stops with two-way ANOVA showed a significant main effect of time [*p* = 0.0044, *F*(4, 48) = 4.358], no significant effect of VPA treatment [*p* = 0.0772, *F*(1, 12) = 3.735] and significant time × VPA interaction [*p* = 0.0036, *F*(4, 18) = 5.784]. Even if no statistically significant difference was found between the control and VPA-group at any minute, the first minute in the control was statistically different from minute five in the same control condition (*p* < 0.05, Tukey *post hoc* test).

On the other hand, minute-by-minute “distance/stop” showed a profile inverse to that of stops, which was expected considering no changes in traveled distance (Figure 1J). “Distance/stop” was relatively stable during the 5 min of the test in the VPA group, while the control group started with an increased value in minutes 1 and 2, which then went down to control values in minutes 3–5. The minute-by-minute analysis of “distance/stop” with two-way ANOVA showed a significant main effect of time [*p* = 0.0002, *F*(4, 48) = 6.892], a significant effect of VPA treatment [*p* = 0.0368, *F*(1, 12) = 5.517] and significant time × VPA interaction [*p* = 0.0474, *F*(4, 18) = 2.979]. There was no statistically significant difference between the control and VPA-group in any minute, but the first minute in the control group was statistically different from minute five in the same control condition (*p* < 0.05, Tukey *post hoc* test).

These data evidenced that prenatal VPA treatment specifically affects the acceleration of movements without influencing the “traveled distance” and “effective velocity” parameters. In addition, habituation to the OFT arena occurred normally at the level of “traveled distance” and only the VPA-group showed an habituation-like behavior at the level of “mean acceleration,” suggesting that not all kinematic parameters show a similar variation as a function of time. Interestingly, the parameters “stops” and “distance/stop” showed a constant behavior in the VPA-group, while the control group was able to modulate them along the time of the test, suggesting that VPA animals decrease in-time acceleration by decreasing the traveled distance but not increasing the stops.

**FIGURE 2:**
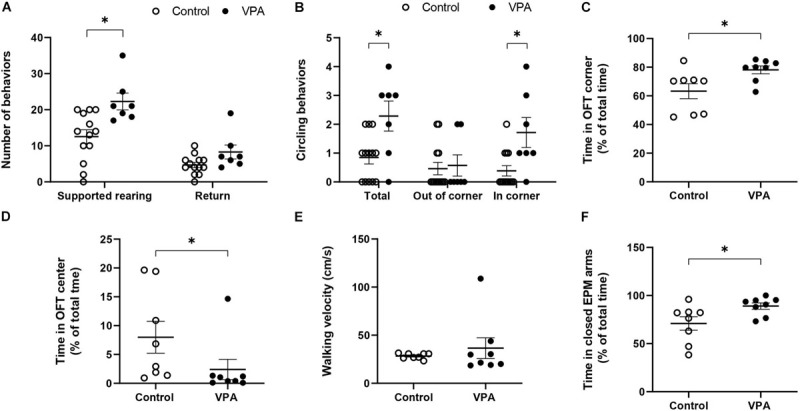
Effect of prenatal VPA treatment on the **(A)** supported rearing (s. rearing) and returns and **(B)** circling activity in the first batch of animals (n controls = 13 and n VPA = 7). **(C)** % of time spent in OFT corners, **(D)** % of time spent in OFT center, **(E)** walking velocity on OFT, and **(F)** % of time in the closed arms of the EPM for the second batch of animals (n controls = 8 and n VPA = 8). ^∗^*p* < 0.05; Mann-Whitney *U*-test, VPA compared to controls.

In order to further analyze the effect of prenatal VPA-treatment in movement characteristics, we quantified particular behaviors, such as “supported rearing” and “circling.” Figure 2A shows that the VPA-group performed nearly twice more “supported rearing” events than the control group (12.54 ± 2 in control vs. 22.3 ± 2.53 in VPA rats, *p* < 0.01, Mann-Whitney *U*-test), without a significant effect on “returns.” Circling activity increased nearly three times in the total OFT arena (regardless of direction) in the VPA-group (0.85 ± 0.23 in control vs. 2.29 ± 0.56 in VPA rats, *p* < 0.05, Mann-Whitney *U*-Test) and was more notorious in the OFT corners (0.38 ± 0.19 in control vs. 1.71 ± 0.56 in VPA rats, *p* < 0.05, Mann-Whitney *U*-Test) (Figure 2B). In addition, VPA rats showed greater permanence in the corners. The time spent in the corner of the OFT arena for VPA rats was lightly superior but the difference was statistically significant (63.26 ± 5.6% in control vs. 78.11 ± 2.9% in VPA group, *p* < 0.05, Mann-Whitney *U*-test) (Figure 2C). Regarding the% of the time spent in the center of the OFT, VPA rats showed a statistically significant (*p* < 0.05, Mann-Whitney *U*-test) lower time (2.41 ± 1.9%) compared to control group (7.99 ± 3%) (Figure 2D).

On the other hand, considering that the kinematic parameters of distance, velocity and acceleration that we calculated involve movements that imply displacement, but not necessarily a particular type of movement, we conducted a preliminary evaluation of walking velocity. For this, we visually selected video segments in which the animal performed a straight-line walk and thus, calculated a velocity that truly represented walking velocity. No significant difference was found in the mean walking velocity between control and VPA rats (28.33 ± 1.11 in control vs. 36.5 ± 11.5 in VPA rats, *p* = 0.43, Mann-Whitney *U*-Test) (Figure 2E). A remarkable observation during this evaluation was the extreme difficulty in finding video segments in which VPA rats walked in a straight line, and the great variability of the data for this group. In contrast, the control group showed minimal variability and many segments of straight-line walking. Of note, given that the results represent mean ± standard error and that the statistical n value was 8 independent individuals for each group, increased variability in the VPA-group was not due to the number of animals.

Since anxiety is a fundamental sign in ASD individuals and in animal models of autism, we conducted the EPM test in order to corroborate this characteristic. Figure 2F shows that the VPA-group spent more time in the closed arms than the control group (71 ± 7.5% in control vs. 89 ± 3.6% in VPA rats, *p* < 0.05, Mann-Whitney *U*-Test) demonstrating a higher level of anxiety in VPA-treated rats.

### Kinematic Parameters Determined During the Habituation Stage in the 3C-ST

**FIGURE 3:**
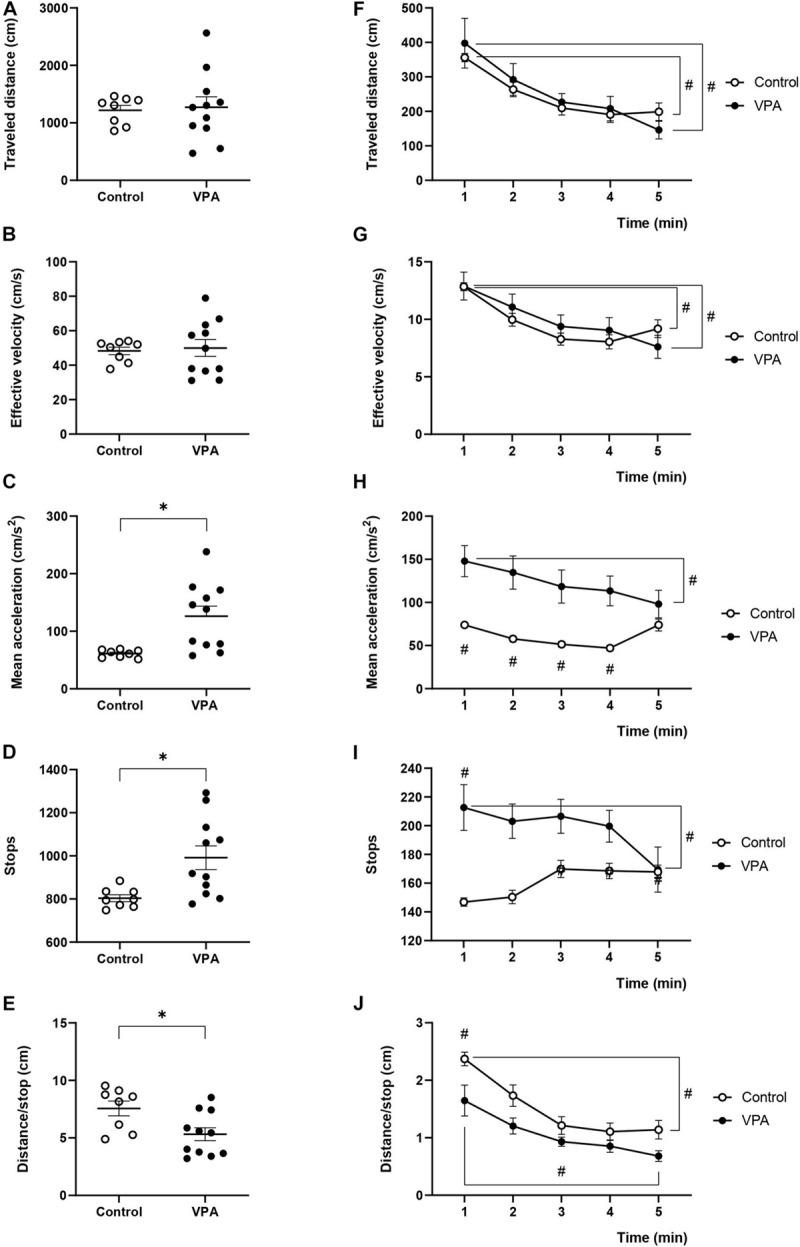
Effect of prenatal VPA treatment on kinematic parameters in the habituation stage of 3C-ST. **(A)** “Traveled distance,” **(B)** “effective velocity,” **(C)** “mean acceleration,” **(D)** “stops,” and **(E)** “distance/stop” in the total time of the habituation stage of the 3C-ST. ^∗^*p* < 0.05; Mann-Whitney *U*-test, VPA compared to controls. Minute-by-minute analysis of the **(F)** “traveled distance,” **(G)** “effective velocity,” **(H)** “mean acceleration,” **(I)** “stops,” and **(J)** “distance/stop.” ^#^*p* < 0.05; Two-way ANOVA, followed by the Tukey *post hoc* test. n controls = 8 and n VPA = 11.

In order to investigate if the kinematic parameters evaluated in the OFT were dependent on the space used in the determination, we analyzed the first stage of the 3C-ST, which lasts 5 min and represents a sort of “open-field” of little dimensions. In fact, we essentially replicated the previous results for the “traveled distance,” “effective velocity,” and “mean acceleration” from the OFT. No significant differences were found between control and VPA groups in “traveled distance” and “effective velocity” during the entire habituation stage of the test (Figures 3A,B). However, the “mean acceleration” in the control group (61.2 ± 2.5 cm/s^2^) significantly increased in the VPA group (126 ± 18.5 cm/s^2^) (*p* < 0.01, Mann-Whitney *U*-test) (Figure 3C).

Total stops in the control rats were 803 ± 17 during the habituation stage of the 3C-ST, and significantly increased to 991 ± 49 in the VPA-group (*p* < 0.01, Mann-Whitney *U*-test) (Figure 3D). Inversely, while the control group traveled a mean of 7.56 ± 0.7 cm between halts, the VPA-group traveled 5.32 ± 0.72 cm in average, which was statistically lower (*p* < 0.05, Mann-Whitney *U*-test) (Figure 3E).

The minute-by-minute two-way ANOVA analysis of “traveled distance” showed a main effect of time [*p* < 0.0001, *F*(4, 40) = 19.25], without an effect of VPA treatment [*p* = 0.8113, *F*(1, 10) = 0.06007] or time × VPA interaction [*p* = 0.2972, *F*(4.25) = 1.300]. We observed a statistically significant gradual decline for “traveled distance” from the first to the last minute in control and VPA groups (*p* < 0.0001 for the VPA-group and *p* < 0.05 for the control group; Tukey *post hoc* test, first minute vs. the last minute in each group, Figure 3F). Moreover, “effective velocity” showed a similar behavior according to two-way ANOVA analysis; main effect of time [*p* < 0.0001, *F*(4, 40) = 22.02], no effect of VPA treatment [*p* = 0.7246, *F*(1, 10) = 2.842], and significant minute × treatment interaction [*p* = 0.0453, *F*(2, 25) = 2.842]. There were no statistical differences between the control and VPA groups in each minute, but a statistically significant decline in the control group (*p* < 0.01; Tukey *post hoc* test, first minute vs. the last minute) and the VPA-group (*p* < 0.0001; Tukey *post hoc* test, first minute vs. the last minute) (Figure 3G). This behavior was different from that observed with the OFT, where the decline from minutes 1 to 5 was not statistically significant in both groups (compare Figure 3G with Figure 1G).

The analysis of “mean acceleration” by two-way ANOVA indicated a significant main effect of time [*p* < 0.0001, *F*(4, 40) = 11.97], VPA treatment [*p* = 0.0134, *F*(1, 10) = 8.973] and time × VPA interaction [*p* < 0.0001, *F*(4, 25) = 9.442]. “Mean acceleration” was relatively stable during the 5 min of the 3C-ST habituation stage in the control group. On the other hand, the VPA-group showed a statistically significant decline (*p* < 0.0001; Tukey *post hoc* test) from the first minute (147.9 ± 18.8 cm/s^2^) to the last minute (98.1 ± 16.8 cm/s^2^), reaching values that were not statistically different from that of the last minute in the control group (Figure 3H). This behavior was completely equivalent to that found in the OFT (Figure 1H).

Regarding the behavior of “stops” during the 3C-ST habituation stage, we observed that the stops increased in the VPA-group compared to the control group in the first minute and a gradual decline to converge to control values was produced in minute five (Figure 3I). Two-way ANOVA indicated no significant main effect of time [*p* = 0.1117, *F*(4, 40) = 2.009], a significant effect of VPA treatment [*p* = 0.0270, *F*(1, 10) = 6.699] and a significant time × VPA interaction [*p* < 0.0030, *F*(4, 25) = 5.345]. Minute five of the VPA-group was significantly lower than the first minute in the same group (*p* < 0.001; Tukey *post hoc* test).

The “distance/stop” parameter showed an inverse pattern respect to the “stops.” We observed decreased “distance/stop” in the VPA-group compared to the control group in the first minute and a gradual decline -also observed in controls-, to converge to control values in minute five (Figure 3J). Two-way ANOVA indicated no significant main effect of time [*p* = 0.1117, *F*(4, 40) = 2.009], a significant effect of VPA treatment [*p* = 0.0270, *F*(1, 10) = 6.699] and a significant time × VPA interaction [*p* < 0.0030, *F*(4, 25) = 5.345]. The differences between the first minute and the last minute in control and VPA groups was statistically significant (*p* < 0.0001 for the control and *p* < 0.0001 for the VPA-group; Tukey *post hoc* test).

In general, the results obtained in the habituation stage of 3C-ST validate our analysis performed with the OFT, and show that prenatally VPA-treated rats do not display considerable differences in their kinematic characteristics (such as distance and velocity). However, a general increase in mean acceleration was present in all conditions, along with increased halts during trajectories and reduced distance between stops, more or less evident depending on the behavioral test used. In fact, increased “stops” and reduced “distance/stop” were more evident in the beginning of the OFT and habituation stage of the 3C-ST.

### Kinematic Parameters Determined During the Sociability Stage of the 3C-ST

**FIGURE 4:**
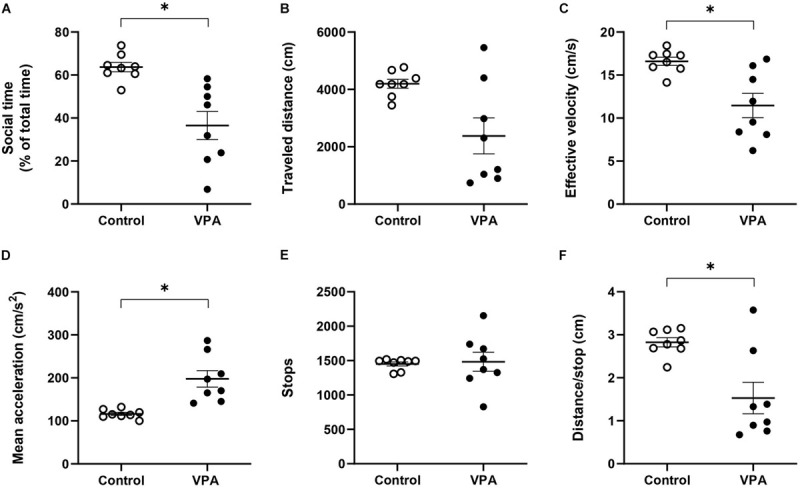
Effect of prenatal VPA treatment on **(A)** social time and kinematic parameters in the social stage of the 3C-ST; **(B)** “traveled distance,” **(C)** “effective velocity,” **(D)** “mean acceleration,” **(E)** “stops,” and **(F)** “distance/stop”. ^∗^*p* < 0.05; Mann-Whitney *U*-test, VPA compared to controls. n controls = 8 and n VPA = 8.

The sociability stage of the 3C-ST lapses for 10 min and in this case we also evaluated kinematic parameters, but now in an alternant social/non-social context. First, we analyzed the percentage of time spent in the social area, corresponding to the chamber where a congener was located. This parameter is usually used as a sign of social preference and its reduction is interpreted as an index of social deficit. While control rats spent 63.7 ± 2.34% of the total time in the social area -showing their natural social preference- the VPA-group spent significantly less time in this area (36.5 ± 7%, *p* < 0.001; Mann-Whitney *U*-test) (Figure 4A). Hence, the VPA-model exhibited social deficits, so we then proceeded to analyze kinematic parameters that differentiated total (all chambers), social (only in the social chamber) and non-social (center plus empty chamber) areas.

First, at the level of total area, we did not observe statistical differences between the control and VPA groups in “traveled distance,” but a reduction tendency in the VPA-group, compared to the control group (*p* = 0.064; Mann-Whitney *U*-test, Figure 4B), probably underlying a statistically significant reduction in “effective velocity” in that group (16.6 ± 0.5 cm/s in the control group vs. 11.5 ± 1.5 cm/s in the VPA-group, *p* < 0.05; Mann-Whitney *U*-test, Figure 4C). Similarly to the OFT and the habituation stage of the 3C-ST, there was a statistically significant increase of “mean acceleration” (p < 0.001; Mann-Whitney *U*-test); from 116.3 ± 3.8 cm/s^2^ in the control group to 197.7 ± 20.5 cm/s^2^ in the VPA-group (Figure 4D). No statistical difference was found at the level of “stops” in the entire area of the 3C-ST social stage (Figure 4E). However, a significant reduction was observed in the “distance/stop” parameter (*p* < 0.05; Mann-Whitney *U*-test) (Figure 4F). These results show that when facing a social condition, acceleration remains elevated, but an additional difference between control and VPA rats appears: reduced velocity, considering all the areas of the 3C-ST.

**FIGURE 5:**
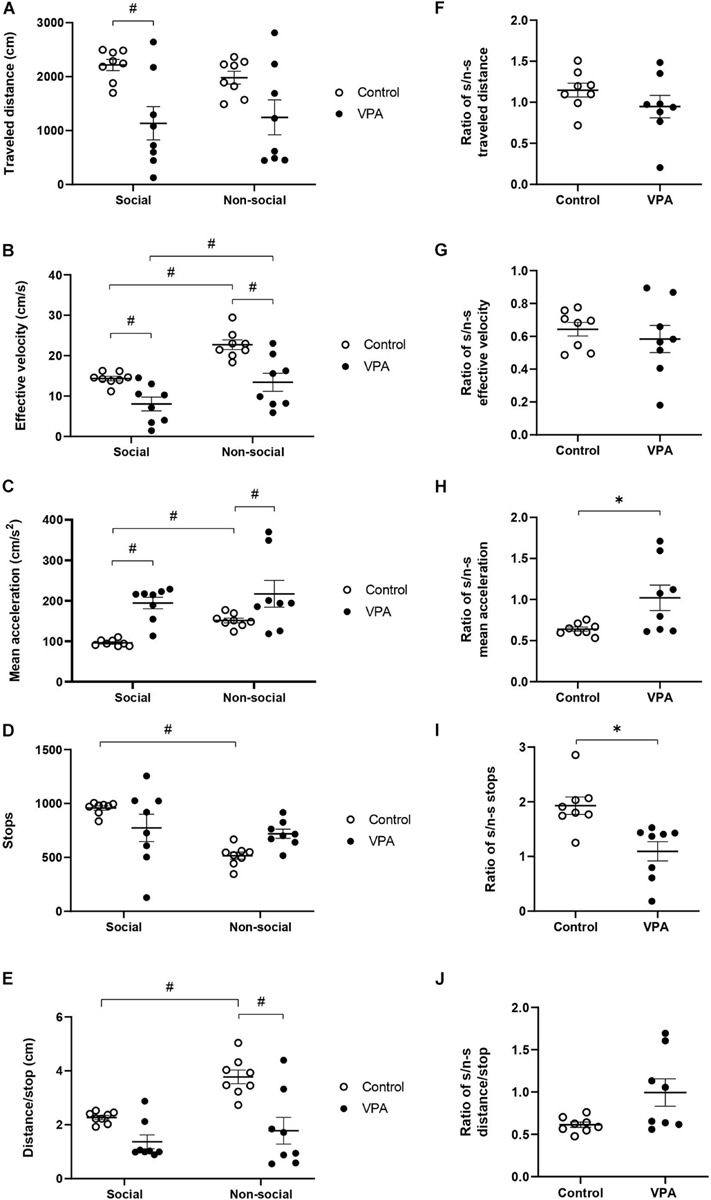
Effect of prenatal VPA treatment on kinematic parameters in social and non-social areas during the social stage of the 3C-ST; **(A)** “traveled distance,” **(B)** “effective velocity,” **(C)** “mean acceleration,” **(D)** “stops,” and **(E)** “distance/stop.” ^#^*p* < 0.05; One-way ANOVA, followed by Sidak *post hoc* test. Social/non-social (S/n-s) ratios of (**F**) “traveled distance,” **(G)** “effective velocity,” **(H)** “mean acceleration,” **(I)** “stops,” and **(J)** “distance/stop.” ^∗^*p* < 0.05; Mann-Whitney *U*-test, VPA compared to controls. n controls = 8 and n VPA = 8.

Furthermore, the analysis of kinematic parameters to separate social from non-social areas showed a differential effect of VPA treatment. First, two-way ANOVA analysis of “traveled distance” showed no effect for area [*p* = 0.4944, *F*(1, 14) = 0.4923], but a significant effect of treatment [*p* = 0.013, *F*(1, 14) = 7.907], without area × treatment interaction [*p* = 0.0741, *F*(1, 14) = 3.725]. The VPA-group showed a reduced “traveled distance” in the social area compared to the control group (2215.27 ± 109.85 cm in controls vs. 1134 ± 329.66 cm in the VPA-group, *p* < 0.01; Sidak *post hoc* test), while no significant difference was found in the non-social area (1079.9 ± 126.9 cm in controls vs. 1244.6 ± 346.05 cm in the VPA-group, p = 0.07; Sidak *post hoc* test) (Figure 5A). In addition, no statistical difference was found in “traveled distance” between social and non-social areas in the control (*p* = 0.1608; Sidak *post hoc* test) or VPA groups (*p* = 0.6397; Sidak *post hoc* test). These results suggest that VPA-treated rats reduce their locomotor activity in the new condition; however, this parameter did not differentiate between social and non-social areas, in both control and VPA groups.

In relation to “effective velocity,” two-way ANOVA analysis showed a significant main effect of area [*p* < 0.0001, *F*(1, 14) = 57.01] and VPA treatment [*p* = 0.0016, *F*(1, 14) = 15.34], without area × treatment interaction [*p* = 0.1200, *F*(1, 14) = 2.742]. A statistically significant reduction was observed in the VPA-group in social (*p* = 0.0153) and in non-social (*p* = 0.0004) areas, in relation to the control group (Sidak *post hoc* test) (Figure 5B). Moreover, both control and VPA groups showed a significant difference between social and non-social areas. The control group showed an “effective velocity” of 14.34 ± 0.6 cm/s in the social area and 22.73 ± 1.3 cm/s in the non-social area (*p* < 0.0001; Sidak *post hoc* test), while the VPA group showed an “effective velocity” of 8.05 ± 1.8 cm/s in the social area and 13.42 ± 2.34 cm/s in the non-social area (*p* = 0.0019; Sidak *post hoc* test). Therefore, “effective velocity,” in contrast to “traveled distance” is a parameter that discriminates between social and non-social contexts in both control and VPA-treated rats.

We then analyzed “mean acceleration” and found that it was the only variable that dramatically differentiated VPA from control animals in “open field” conditions (OFT and the habituation-stage of the 3C-ST). Two-way ANOVA analysis indicated a main effect of area [*p* = 0.0253, *F*(1, 14) = 6.265] and VPA treatment [*p* = 0.0014, *F*(1, 14) = 15.69], without area × VPA interaction [*p* = 0.3133, *F*(1, 14) = 1.094]. A statistically significant increase of “mean acceleration” was observed in the VPA-group in both social (*p* < 0.01) and non-social (*p* < 0.05) areas, in relation to the respective areas in the control group (Sidak *post hoc* test) (Figure 5C). While acceleration in the social area was 98 ± 4.8 cm/s^2^ in the control group, the VPA-group showed a value of 194 ± 15.4 cm/s^2^. In the non-social area, control acceleration was 151.2 ± 6.3 cm/s^2^, increasing to 217.1 ± 35.4 cm/s^2^ in the VPA-group. On the other hand, social vs. non-social areas presented a statistically significant difference in the control group (*p* < 0.001; Sidak *post hoc* test), but not in the VPA-group (*p* = 0.8200) (Figure 5C), suggesting that in the control group, mean acceleration is able to discriminate social from non-social areas, while the VPA group loses that ability.

“Stops” in social and non-social areas also showed a differential behavior between control and VPA groups. Two-way ANOVA analysis of stops indicated a main effect of area [*p* = 0.0024, *F*(1, 14) = 13.66], no effect of VPA-treatment [*p* = 0.9002, *F*(1, 14) = 0.01632] and a significant area × VPA interaction [*p* = 0.0120, *F*(1, 14) = 8.326] (Figure 5D). A statistically significant difference was found between social and non-social areas in the control group (*p* = 0.0007), but not in the VPA group (*p* = 0.8200; Sidak *post hoc* test). However, no significant difference was observed between control and VPA groups in social (*p* = 0.1368) or in non-social (*p* = 0.0944) areas (Sidak *post hoc* test). This result suggests that the control group is able to discriminate social from non-social areas in this parameter, while the VPA-group loses that ability.

On the other hand, two-way ANOVA analysis of “distance/stop” showed a main effect of area [*p* < 0.0001, *F*(1, 14) = 32.29] and VPA treatment [*p* = 0.0029, *F*(1, 14) = 13.00] and a significant area × VPA interaction [*p* = 0.0058, *F*(1, 14) = 10.55] (Figure 5E). The difference between social and non-social areas in the control group was statistically significant (*p* < 0.0001), but not for the VPA-group (*p* = 0.2031; Sidak *post hoc* test). No significant difference of distance/stop was observed in the social (*p* = 0.0940) area, but a significant difference was found in the non-social area when comparing the control with the VPA-group (*p* = 0.0002; Sidak *post hoc* test), suggesting that this parameter discriminates social from non-social areas in controls, but not in VPA treated rats.

To corroborate the specificity of the kinematic parameters in the loss of social/non-social discrimination, we calculated a social/non-social ratio for the kinematic variables. In the control group, the social/non-social ratio for distance was 1.15 ± 0.09, and it was not statistically different from the VPA-group, which showed a ratio of 0.95 ± 0.16 (*p* = 0.16; Mann-Whitney *U*-test, Figure 5F), suggesting no context discrimination for this parameter in control and VPA groups. On the other hand, the social/non-social ratio for “effective velocity” was similar in the control group (0.64 ± 0.04) to that of the VPA-group (0.54 ± 0.08), suggesting that both groups decrease their velocity in the social area in a similar way (*p* = 0.64; Mann-Whitney *U*-test, Figure 5G); thus, this parameter is able to discriminate social from non-social contexts in both groups. In contrast, the social/non-social ratio for “mean acceleration” was significantly higher in the VPA-group (1.05 ± 0.18) than in the control group (0.64 ± 0.03) (*p* < 0.05; Mann-Whitney *U*-test, Figure 5H). The social/non-social ratio for “stops” was 1.93 ± 0.1 in the control group, showing a preference for halting in the social chamber, while the VPA-group ratio was 1.09 ± 0.2, suggesting no preference (Figure 5I). The difference between social/non-social ratios for stops was statistically significant (*p* = 0.030, Mann-Whitney *U*-test), suggesting that controls discriminate by context, an ability lost in the VPA-group. Regarding “distance/stop,” no statistical difference was found between control and VPA groups (*p* = 0.0830, Mann-Whitney *U*-test) (Figure 5J).

The results obtained in the social stage of the 3C-ST corroborated increased acceleration in the VPA group and suggests that control animals reduce their movement acceleration and increase their halts when they move in the social chamber, while VPA-treatment abolishes the ability to modulate acceleration and halts when the rats face social or non-social contexts.

## Discussion

We have studied basic kinematic parameters in the juvenile rat VPA-model of autism using two behavioral tests (OFT and 3C-ST), which represent two different contexts. The most remarkable observation of our work was evidenced in the social stage of the 3C-ST, where, according to the “mean acceleration” and “stops” parameters, the social/non-social context discrimination was lost in the VPA-group, whereas distance does not discriminate a social context, while velocity does in both groups. In addition, we showed that VPA treatment produced consistent increases in “mean acceleration” and reduced “distance/stop,” with almost null effect on “traveled distance” and “effective velocity” in the OFT, an effect corroborated during the habituation stage of the 3C-ST. However, in the last case, we observed additionally increased “stops.” These results suggest that in a socially neutral context, the motor alterations in ASD may specifically affect certain kinematic parameters; i.e., acceleration and related variables as “stops” and “distance/stop,” without evidently affecting distance and velocity. Moreover, in a condition where there is an alternation of social vs. non-social contexts, velocity is also affected and more importantly, we evidenced an effect in the ability to dynamically change “mean acceleration” and “stops” parameters between social and non-social contexts. To our knowledge, this is the first report in an animal model of autism that shows this type of discriminative behavior for acceleration and stops variables in control rats, and its loss in VPA-treated rats.

In the OFT, control and VPA-treated rats exhibited similar kinematic characteristics, such as total displacement and velocity. In addition, according to the “traveled distance” parameter, both groups seemed to equally habituate to the arena. In order to obtain a real parameter of movement velocity in general, but not associated with a particular type of movement (walking, jumping, turns, etc.), we calculated “effective velocity” as the mean velocity, considering only the time in which the animal showed a displacement of its center of mass, respect to the precedent video frame. Furthermore, despite the similarity displayed by control and VPA groups in these two basic kinematic parameters, we found an important difference in movement acceleration, measured as “mean acceleration.” Nonetheless, it is important to mention that we cannot infer whether these differences are associated with a particular movement characteristic; in other words, related to increased acceleration, which might represent less smooth movements or faster movement initiation. Even though a more precise kinematic study is necessary to clear this point out, we can conclude that the observed differences represent an intrinsic characteristic of the animal movements affected by the VPA treatment. A previous report in which kinematic parameters were studied in different strains of control mice has indicated that the acceleration-influenced parameter “velocity/stops,” may differentiate mice strains, and this parameter was associated with “darting behavior,” which can reflect vulnerability to stress, anxiety or jitteriness (de Visser et al., 2006). In fact, we performed the elevated-plus maze and found evidence of increased anxiety in VPA-treated rats, a feature also corroborated by the reduced time in the center of OFT arena in our experimental group. Hence, the increased acceleration that we found in the VPA-group, in addition to conserved or reduced velocity could be concordant with the recognized anxious profile characteristic of this ASD animal model (Markram et al., 2008; Markram and Markram, 2010).

Of note, VPA-treated animals normally habituate the “traveled distance” parameter in the OFT and the 3C-ST, while “mean acceleration” reflects a habituation-like behavior only in VPA animals. In addition, velocity showed a habituation-like behavior in both control and VPA rats in the little arena of habituation stage of 3C-ST. We suggest that acceleration is a more sensitive parameter for detecting anxiety in VPA animals. In fact, Olexová et al. (2013), measured displacement and showed that VPA-treatment reduces habituation, but during a longer period of time (15–20 min). It is interesting that we found increased “stops” and decreased “distance/stop” in the first minute of the OFT and in the habituation stage of 3C-ST, but these parameters reached control values at the second or third minute of the test, suggesting that “stops” and “distance/stop” could be related to “acceleration.”

Since our kinematic parameters did not characterize any particular type of movement, we also evaluated walking, rearing, turns and circling behavior. When comparing control and VPA rats, we did not find any differences in walking velocity, but we had difficulties in finding video sequences in which VPA rats walked in a straight line, reflecting their propensity to perform circling behavior, especially in the corners of the arena test, where they also spent more time in comparison to controls. Increased circling behavior and supported rearing may suggest anxious behavior but, considering that standing upright is a normal behavior (Makowska and Weary, 2016), we cannot discard the idea that these corner-directed behaviors may reflect a particular type of exploration induced by VPA treatment. Circling behavior has already been reported in another animal model, in which VPA administration is performed early in postnatal (PN4-PN11) mice (Bath and Pimentel, 2017). Interestingly, a particular type of circling motor activity has been described in ASD children and was reported as “stereotypical spinning behavior” (Bracha et al., 1995) or a “turning bias,” which correlates with ASD severity (Cohen et al., 2014). Another report, using a fully automatic system, showed greater and faster head turning in ASD children (Martin et al., 2018). Hence, the circling activity displayed by the VPA-group seems to represent a behavioral characteristic of ASD subjects, which can be objectively measured in both human and animal models. Furthermore, atypical movements have been reported in different studies. For example, one report associated increased acceleration with increased velocity and “jerky” arm movements in ASD individuals (Cook et al., 2013). On the other hand, increased acceleration has been associated with oscillations of the trunk and head during gait, and determines slow walking in ASD adults (Armitano et al., 2020). These observations resemble our findings of reduced velocity associated with increased acceleration in the social stage of the 3C-ST, and may be related to the reduced distance traveled between halts, also observed in our study.

Our findings might be explained by alterations of several neurobiological substrates, including several supra-spinal motor control structures. The method that we used to calculate the kinematic parameters does not address a particular type of movement, but considering that it is based on the displacement of the animal’s center of mass, locomotion may be a main component influencing our results. It is well recognized that the mesencephalic locomotor region (LMR), located in the upper brainstem, is fundamental in defining walking velocity (Skinner and Garcia-Rill, 1984; Sherman et al., 2015), although the LMR may interact with certain neuronal populations located in the caudal brainstem (Capelli et al., 2017). Interestingly, brainstem structural alterations were the first morphological hallmarks described in both, the VPA model, and in *postmortem* encephalic tissue from ASD individuals (Rodier et al., 1996; Inui et al., 2017). Moreover, locomotor nuclei in the LMR can be classified into areas associated with exploratory, appetitive, and defensive locomotion, which interact with frontostriatal circuits of the basal ganglia, lateral and medial hypothalamus, respectively (Jordan, 1998).

Based on the exploratory component of our behavioral tests and taking into account the particular exploratory behavior displayed by VPA-treated rats in the OFT arena, basal ganglia circuits may be interesting regions in which the neurobiological underpinnings of the kinematic alterations observed in ASD could be further investigated. In fact, multiple alterations have been described in basal ganglia circuits in ASD individuals and animal models of autism (Subramanian et al., 2017). This collection of subcortical nuclei, connected reciprocally with the frontal cortex, is associated with a number of motor, associative and cognitive functions that involve several complex behaviors, such as joint attention, imitation, communication, inhibitory control, action chaining, compulsive and goal-directed behaviors, which are all altered in ASD (Subramanian et al., 2017). Furthermore, among the most relevant neurotransmitters of the basal ganglia is dopamine (DA), a neurotransmitter produced by the mesencephalic nigral neurons and critically involved in exploratory behavior (Alexander and Crutcher, 1990). The similarity between the motor alterations produced in ASD, Parkinson’s disease and PANDAS (pediatric autoimmune neuropsychiatric disorder associated with streptococcal infections), where dopaminergic neurons are targeted, suggest a pivotal role for DA in ASD pathophysiology (Paval, 2017). Hence, the particular exploratory behavior and altered kinematics reported in our study in VPA rats, including the loss of social context discrimination, might reflect dopaminergic dysfunction of basal ganglia circuitry produced by the embryonic VPA-treatment. In agreement with this idea, altered dopaminergic neurotransmission has been reported in the VPA-model, characterized by reduced DA release in the prefrontal cortex and attenuated hyperlocomotive response to a methamphetamine challenge (Hara et al., 2015). However, it seems that DA has mixed actions because an increased baseline DA overflow level has been reported in the frontal cortex of VPA-treated rats (Nakasato et al., 2008). More investigations are necessary to elucidate de precise relationship between dopaminergic dysfunction and the motor and kinematic alterations observed in ASD.

Kinematic studies in ASD are scarce but, an interesting work showed a characteristic kinematic signature regarding micro-movements during a reaching task in ASD (Torres et al., 2013). The authors found that, while goal-directed segments of movement are clearly distinguished from spontaneous segments in control people, ASD individuals did not showed such difference. Studying the variability of such micro-movements, the same authors suggest that ASD subjects do not acquire the motor performance amelioration that spontaneously arises from autonomous motor exploration, suggesting a difficulty in the flexible switching of behavior. Our data, although obtained from a completely different method of kinematic analysis, also suggest that behavioral inflexibility is expressed, or generated, at the most basic level of motor control.

An interesting recent report of Vabalas et al. (2020) used machine learning-data mining on eye-tracking and hand-movement tracking data from a control/ASD sample to predict ASD in another independent sample, with a success of 70–78%. These results support the use of kinematic analysis as a useful tool to aid in ASD diagnosis and reinforce the importance of kinematic features as distinctive parameters of ASD. Interestingly, the authors comment, as a main observation, that control individuals modulated acceleration/deceleration and vertical amplitude in order to copy unusual elevated movement kinematics, while autistic individuals tended to retain their usual style of movement. These results agree with the idea that behavioral inflexibility in ASD is observed even at the basic level of motor control. Further examples supporting the utility of movement analysis to aid in ASD diagnosis or treatment-following can be found in Budman et al. (2019) and Fulceri et al. (2019).

On the other hand, studies in the VPA-model do not normally address kinematic parameters, such as velocity and acceleration. Only a recent report evaluated velocity in control and postnatally VPA-treated rats in a context of empathy-like pro-social behavior, evaluating the willingness of rats to open a restrainer where a conspecific was trapped (Fontes-Dutra et al., 2019). Interestingly, VPA-treated rats took more time when first opening the restrainer, but showed the same percentage of opening from the first day of opening, compared to the control group. The authors suggest that VPA-rats are able to display empathy-like behavior, which is hampered by the abnormal anxiety or deficient communication of these animals. Importantly, they showed that increased average velocity in the entire test-apparatus or in the door zone was a good predictor for the first day of opening, both in the control and VPA groups, without differences in time or distance in the door zone. Even though acceleration was not evaluated in this study, these data suggest that the ability to change a kinematic parameter depending on the social situation could be related to a pro-social behaviour.”

On the other hand, atypical kinematics has been evidenced in ASD individuals by studying a simple sinusoidal arm movement and, importantly, the degree of such atypicality correlates with the ADOS score and the difficulty to interpret biological movement (Cook et al., 2013). Nevertheless, how can a simple alteration in a basic kinematic parameter affect such a complex and high-order function of the brain such as social cognition? An interesting discussion presented by the same authors argues how such a simple kinematic alteration could finally exert a relevant impact on biological movement perception and, thus, on reciprocal social cognition (Cook, 2016). Indeed, the “motor resonance” hypothesis explains how watching another person perform a movement induces activity in one’s own motor areas, a phenomenon mediated by the “mirror neurons” (Zentgraf et al., 2011). Increasing evidence (discussed by Cook, 2016) has shown that movement similarity is fundamental for action perception and the prediction of others’ behavior. In addition, the resulting movement synchronicity and automatic imitation results intrinsically rewarding, promoting positive emotional connections. Hence, atypical kinematics in ASD individuals might reduce motor similarity between autistic and non-autistic people, bidirectionally breaking the virtuous circle of “motor resonance” and thus, dramatically impacting the high order processes of social cognition.

Finally, we provide, for the first time, evidence of the effect of prenatal exposition to VPA -a “*bona-fide*” model of autism- on the social/non-social context discrimination by the kinematic variables, acceleration and stops, suggesting that these specific parameters may reflect behavioral inflexibility, a fundamental alteration of ASD, pervasive even at the most basic levels of motor control.

## Data Availability Statement

The datasets presented in this article are not readily available because data sharing is not applicable to this article as no new data were created in this study. Requests to access the datasets should be directed to EA, ealiaga@ucm.cl.

## Ethics Statement

The animal study was reviewed and approved by the Ethic Committee of the Chilean Science and Technology National Commission (CONICYT).

## Author Contributions

EA and JF designed the study, analyzed and interpreted the data, and wrote the article. NQ, SS-O, and FP performed the experiments, analyzed the data, and participated in the final correction of the article. FA, KM-G, and CM-R participated in the analysis and interpretation of data and editing of the article. All authors gave approval of the final version to be published and agreed to be accountable for all aspects of the work.

## Conflict of Interest

The authors declare that the research was conducted in the absence of any commercial or financial relationships that could be construed as a potential conflict of interest.

## Footnotes

1 https://imagej.nih.gov/ij
